# A novel arthroscopic classification of labral tear in hip dysplasia

**DOI:** 10.1371/journal.pone.0240993

**Published:** 2020-10-22

**Authors:** Pil Whan Yoon, Jun-Ki Moon, Jae Youn Yoon, Sunhyung Lee, Soong Joon Lee, Hee Joong Kim, Chul-Ho Kim

**Affiliations:** 1 Department of Orthopedic Surgery, Asan Medical Center, Ulsan University, College of Medicine, Seoul, Republic of Korea; 2 Department of Orthopedic Surgery, Hanyang University Guri Hospital, Gyomoon-dong, Guri-si, Gyunggido, Republic of Korea; 3 Department of Orthopedic Surgery, Dongguk University Ilsan Hospital, Siksadong, Ilsandonggu, Goyangsi, Gyeonggido, Republic of Korea; 4 Department of Orthopedic Surgery, Seoul National University College of Medicine, Daehak-ro Jongno-gu, Seoul, Republic of Korea; 5 Department of Orthopedic Surgery, Gachon University Gil Medical Center, Namdong-daero beon-gil, Namdong-gu, Incheon, Republic of Korea; Humanitas Clinical and Research Center - IRRCS, ITALY

## Abstract

**Background:**

Acetabular labral tears cause of pain in patients with symptomatic hip dysplasia. To date, no structured grading system has been developed to evaluate labral tears in these patients. The present study describes a new system of grading labral tears in patients with acetabular dysplasia.

**Methods:**

The data of 66 patients who underwent hip arthroscopy for symptomatic hip dysplasia from March 2014 to February 2018 were reviewed. Labral tears were classified into four groups, based on the occurrence of chondrolabral junction (CLJ) disruption, capsulolabral recess (CLR) disruption, and labral displacement. Labral tears without instability were classified as grade 1 or 2. Partial delamination or blistering of the labrum with minimal fraying at the CLJ was classified as grade 1, whereas labral tears with CLJ disruption were classified as grade 2. Unstable labral tears with CLR disruption followed by CLJ disruption, but without labral displacement, were classified as grade 3, whereas unstable labral tears with CLR and CLJ disruption, but with labral displacement, were classified as grade 4. The radiological and clinical characteristics of patients in each grade were determined including by simple radiographs and MRI/MR arthrography, as were concomitant findings, including rupture of the ligamentum teres, articular cartilage damage, and presence of a paralabral cyst. The surgical options selected for each grade and clinical outcomes, including modified Harris hip scores (mHHS) and Western Ontario and McMaster Universities Osteoarthritis index (WOMAC) scores, were evaluated. Spearman’s correlation analyses were performed to assess whether labral tear grade correlated with baseline characteristics, the incidence of concomitant injuries, and the severity of osteoarthritis (OA). The Wilcoxon test for paired data was performed to compare treatment results with pain scores.

**Results:**

The study cohort included six men and 53 women of mean ± SD age 39.9 ± 13.0 years (range, 15–66 years). Of the 66 hips, seven (10.6%), 10 (15.2%), 30 (45.5%), and 19 (28.8%) were classified as grades 1–4, respectively. Symptom duration (*P* = 0.017), preoperative Tönnis OA grade (*P* < 0.001), cartilage damage (*P* < 0.001), and the presence of a paralabral cyst (*P* = 0.001) correlated significantly with baseline tear grade. In all groups, mHHS and WOMAC scores improved after surgical treatment.

**Conclusions:**

Arthroscopic findings of labral tears in patients with hip dysplasia differed from the conventional classification. The classification system proposed in this study will likely be useful for determining the degree of labral tear in patients with hip dysplasia and for predicting treatment outcomes.

## Introduction

Acetabular labral tears are a major cause of pain in patients with symptomatic hip dysplasia. Labral tears are triggered by two main conditions, hip dysplasia and femoroacetabular impingement (FAI). Labral tears in patients with FAI are caused by abnormal and repetitive contact between the femur and the acetabulum, whereas labral tears in patients with hip dysplasia are caused mainly by structural instability and the shear load of the hip joint [1]. Although several studies have addressed the pathomechanism and treatment of labral tears associated with FAI [2, 3], the mechanisms responsible for labral tears in patients with acetabular dysplasia remain unclear.

Several systems have been formulated to classify labral tears [4–7]. For example, hip labral tear has been classified arthroscopically as similar to meniscal injuries of the knee [5]. Similarly, acetabular labral tears were classified in cadavers according to the histological features and microvasculature structure of the labrum [7]. Most recently, the Multicenter Arthroscopy of the Hip Outcomes Research Network (MAHORN) classified labral tears based on their sizes, tear patterns, and intrasubstance changes [6]. However, all of these systems are difficult to apply to the specific tear features observed in patients with hip dysplasia, and it is difficult to address the clinical findings associated with each type of tear. The present study classified labral tears by analyzing those observed intraoperatively in patients with symptomatic hip dysplasia who underwent hip arthroscopy with or without periacetabular osteotomy (PAO). This study hypothesized that 1) the pattern of labral tears in dysplastic hips is specific to this condition; 2) the tear grade in this classification system follows the natural course of the disease and is unrelated to patients’ baseline demographic characteristics; and 3) concomitant injuries, such as tears of the ligamentum teres (LT), damage to hip joint cartilage, and paralabral cysts, as well as their severity, are related to labral tear grade.

## Materials & methods

### Patient selection

The study protocol was approved by the institutional review board at Asan Medical Center (IRB No: 2019–1645) and written informed consent was waived. The study cohort included all patients who underwent hip arthroscopy with or without PAO for symptomatic hip dysplasia at our institution from March 2014 to February 2018. Patients who had undergone previous surgery on ipsilateral hips and those lost to follow-up within 12 months after surgery were excluded.

Hip dysplasia was diagnosed based on patient history, physical examination, and radiographic evaluation. The radiologic inclusion criteria for hip dysplasia were defined as a lateral center edge angle (LCEA) < 20°, a sharp angle > 45°, a Tönnis angle > 14°, or an acetabular depth to width ratio (AD/WR) < 0.25 [8]. All patients underwent standard radiographic examinations, including a standing anteroposterior (AP) view of the pelvis, frog-leg lateral view, and 45° Dunn view. Pelvic radiographs were taken with patients in the supine position with a tube-to-film distance of 102 cm (40 in) and the tube perpendicular to the table. The beam crosshairs were centered on the point midway between the superior border of the pubic symphysis and a line connecting the anterior superior iliac spines. The pelvic AP view was considered true when the coccyx tip and pubic symphysis were in line and the distance between them was between 1 and 3 cm, and when both teardrops, the iliac wing, and the obturator foramen were symmetrical. To determine labral pathology before surgery, the results of intravenous gadolinium-enhanced pelvic MRI or MR arthrography were reviewed in all patients who underwent arthroscopic treatment due to symptomatic hip dysplasia. However, because of the retrospective nature of this study, the MR protocol could not be standardized (of 66 patients, 34 underwent MR arthrography, 20 underwent gadolinium-enhanced MRI in our institution, and 12 underwent MRI at another hospital), preventing pairing of standardized MR images and arthroscopic findings of labral tear grade. The clinical and radiological data were retrospectively obtained from electronic medical records, following approval by the Institutional Review Board of Asan Medical Center (IRB No: 2019–1645).

### Surgical technique

All procedures were performed by two attending surgeons, a specialist in hip arthroscopy with over 10 years of surgical experience, and a specialist in hip preservation surgery with over 25 years of experience in PAO surgery.

Arthroscopy was performed using standard anterolateral, posterolateral, and modified anterior portals, and, if necessary, an additional secured distal-anterolateral accessory portal. The joint was vented during distraction, enabling diagnostic arthroscopy, with all intra-articular pathologies treated arthroscopically. In patients undergoing combination arthroscopy and PAO, hip arthroscopy was performed first, followed by PAO surgery in the same manner as that used for arthroscopy alone. The PAO procedures were performed via dual incision, as described [9].

Labral injuries were managed by consistently following the tear patterns. Fraying lesions were treated by simple debridement. If a labral injury could be repaired, the labrum was sutured with 2.3 mm suture anchors (Smith & Nephew, Andover, MA, USA) using a labral base suture technique [10]. If the tear had progressed extensively and displaced, a loop-around suture technique was used for stability. If labral repair was impossible, the labrum was partially resected.

### Labral tear classification

Labral tears were classified into four grades based on disruptions of the chondrolabral junction (CLJ) and the capsulolabral recess (CLR), as well as on labral displacement, and the instability of the torn labrum as determined arthroscopically.

**Fig 1:**
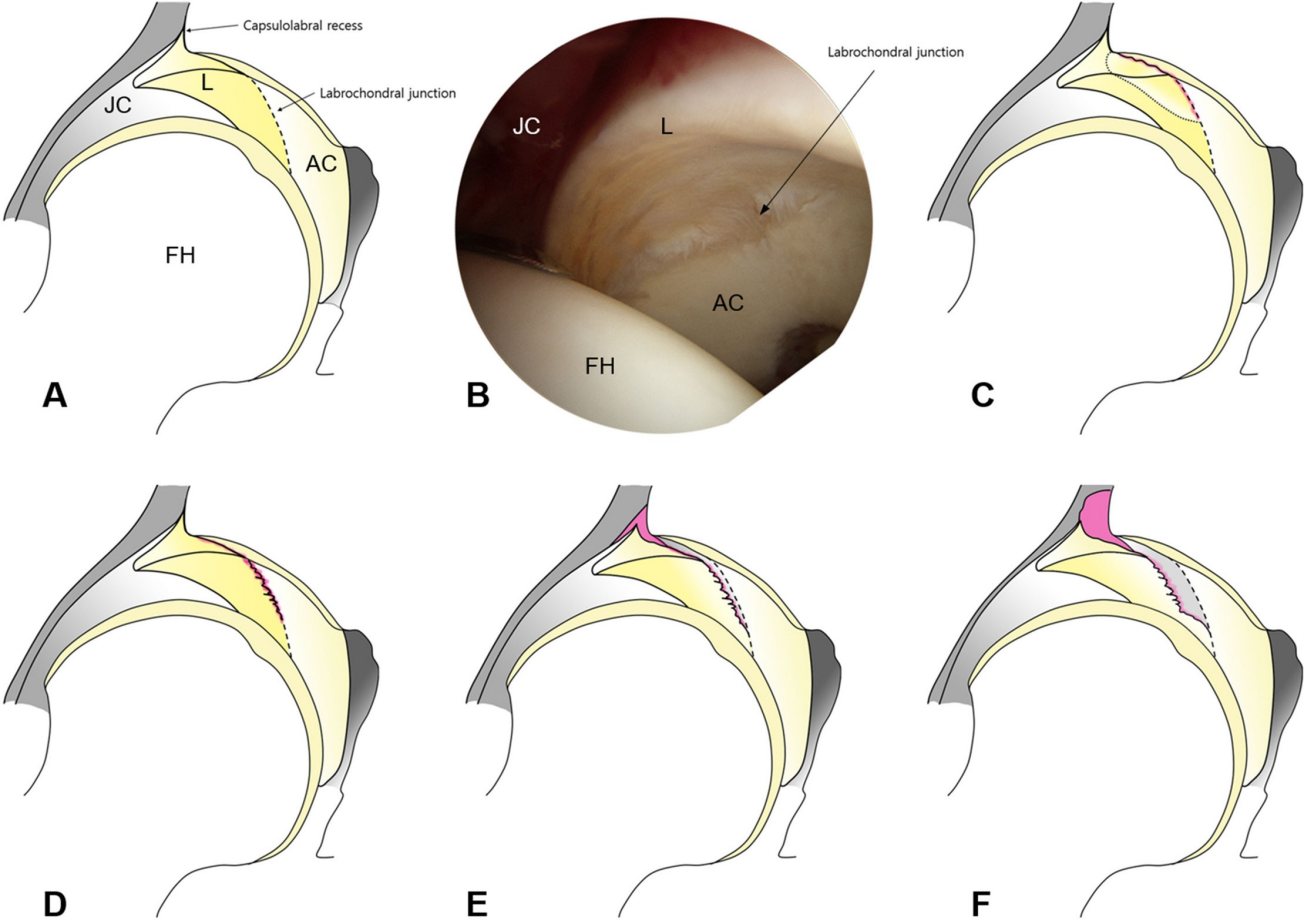
Schematic representation of labral tear grades in patients with hip dysplasia. (A) The left hip joint from behind. (B) Arthroscopic view of the left hip through the anterolateral portal using a 70° arthroscope. (C) Grade 1: partial delamination or blistering of the labrum with minimal fraying at the CLJ. (D) Grade 2: labral tears with CLJ disruption, but no instability. (E) Grade 3: unstable labral tears with CLJ and CLR disruption, but no labral displacement. (F) Grade 4: unstable labral tears with CLJ and CLR disruption, which displaced the labrum laterally. (L, labrum; FH, femoral head; AC, acetabular cartilage).

Labral tears without instability were classified as grade 1 or 2. Partial delamination or blistering of the labrum with minimal fraying at the CLJ was classified as grade 1, whereas labral tears with CLJ disruption were classified as grade 2. Unstable labral tears with CLR disruption followed by CLJ disruption, but without labral displacement, were classified as grade 3, whereas unstable labral tears with CLR and CLJ disruption, but with labral displacement, were classified as grade 4. This classification system is schematically detailed in Fig 1.

### Data collection

To measure interobserver agreement in labral tear grading, grades were determined by the operating surgeon and reviewed by an orthopedist specializing in the hip who did not participate in these operations. The intraoperative arthroscopic images and recorded videos of patients who underwent hip arthroscopy were evaluated by a reviewer who did not participate in the surgical interventions. Labral tear locations were assessed using six acetabular zones [11]. Baseline characteristics measured on plain radiographs of these patients included LCEA, sharp angle, Tönnis angle, and AD/WR. The hip radiographs were reviewed by two experienced orthopedic surgeons. Other characteristics measured included body mass index (BMI) and time from symptom onset to the date of surgery.

Intraoperative arthroscopy was utilized to determine injuries concomitant with labral tears, including LT tears, damage to femoral head (FH) and acetabular cartilage, and the occurrence of paralabral cysts. These findings were compared in groups of patients classified by grade of labral tears. Choice of surgical procedures was also compared in these groups of patients. Modified Harris hip scores (mHHS) and Western Ontario and McMaster Universities Osteoarthritis index (WOMAC) scores measured. Also evaluated at final follow-up were the numbers of patients who required revision hip arthroscopy or conversion hip arthroplasty because treatment failed to stop the progression of osteoarthritis (OA). Tönnis OA grade was also evaluated, not only to evaluate the relationship between labral tear grade and OA severity, but to evaluate the effects of surgical treatment on OA severity at each labral tear grade. All of the clinical data were obtained at admission and at each follow-up visit to the outpatient clinic. The patients were followed-up at 6, 12 weeks and every 6 months thereafter.

### Statistical analysis

Agreement between reviewers was determined by measuring Cohen’s kappa coefficient a priori, with κ = 1 corresponding to perfect, 1.0 > κ ≥ 0.8 to almost perfect, 0.8 > κ ≥ 0.6 to substantial, 0.6 > κ ≥ 0.4 to moderate, 0.4 > κ ≥ 0.2 to fair, and κ < 0.2 to slight agreement. Spearman’s correlation analyses were performed to assess whether labral tear grade correlated with baseline characteristics, the incidence of concomitant injuries, and the severity of OA. The Wilcoxon test for paired data was performed to compare treatment results with pain scores. All statistical analyses were performed using PASW Statistics software, version 18.0 (IBM Corp., Armonk, NY, USA), with *P* < 0.05 defined as statistically significant.

## Results

### Patient demographics

Seventy-three hips underwent hip arthroscopy after initial screening. After excluding three patients who had previously undergone surgery on their ipsilateral hips and four patients who were lost to follow-up within 12 months after surgery, 66 hips were included. Of these, 36 hips underwent hip arthroscopy alone, and 30 underwent PAO combined with hip arthroscopy. Seven patients underwent surgery bilaterally. The cohort included six men and 53 women, of mean ± SD age 39.9 ± 13.0 years (range, 15–66 years) and mean ± SD BMI of 24.3 ± 4.0 kg/m^2^ (range, 18.1–36.0 kg/m^2^). The mean follow-up period was 29.5 ± 11.2 months (range, 12.2 to 51.5 months).

### Arthroscopic classification

**Fig 2:**
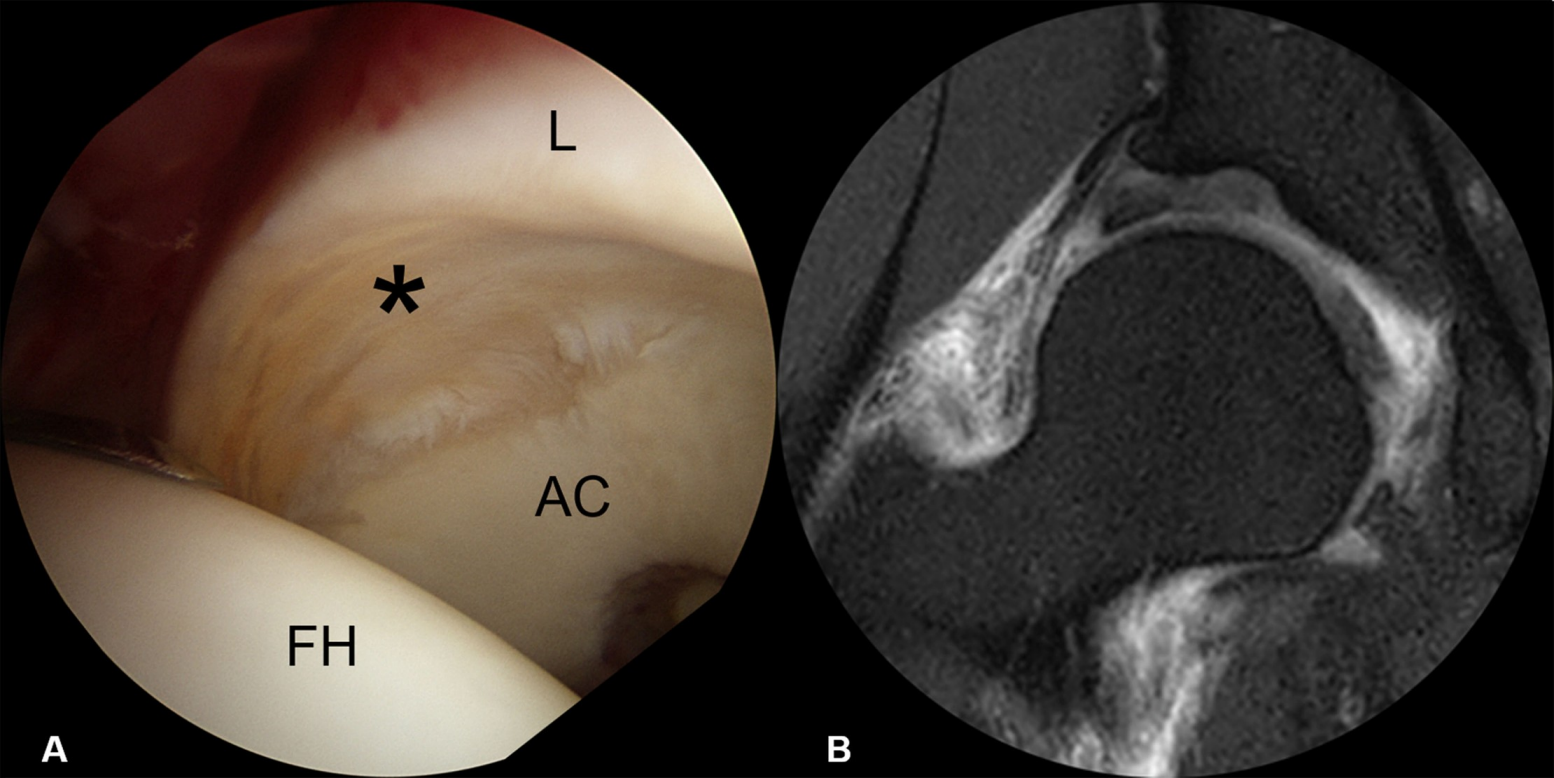
Arthroscopic findings and MRI features of grade 1 labral tears. (A) Arthroscopy showing partial delamination with fraying at CLJ, with (B) MRI showing no definite labral pathology. (L, labrum; FH, femoral head; AC, acetabular cartilage).

**Fig 3:**
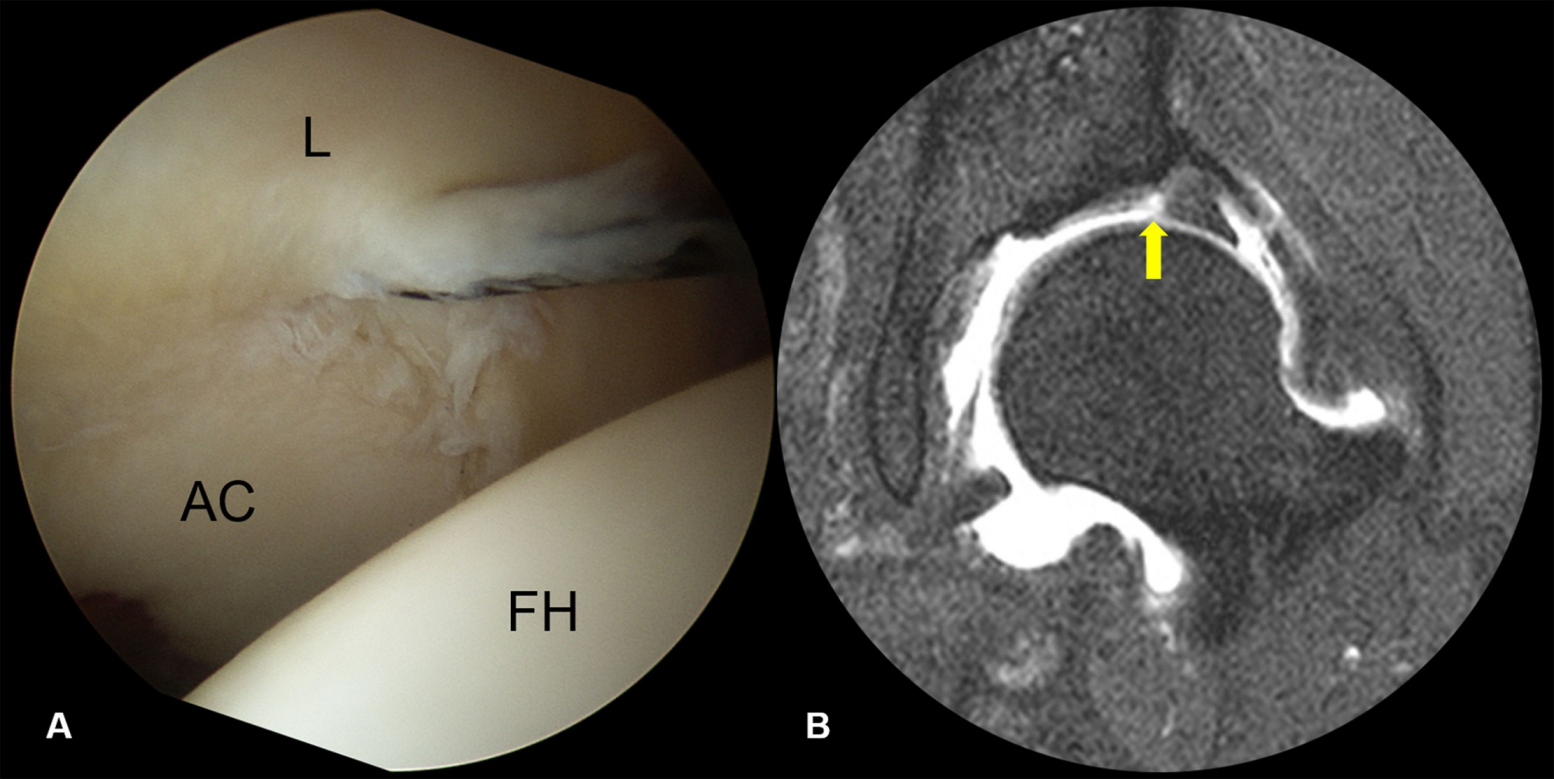
A grade 2 labral tear showing CLJ disruption but stable CLR. (A) Arthroscopy showing a grade 2 labral tear. (B) MR arthrography showing a contrast material-filled defect at the labrochondral junction (arrow). (L, labrum; FH, femoral head; AC, acetabular cartilage).

**Fig 4:**
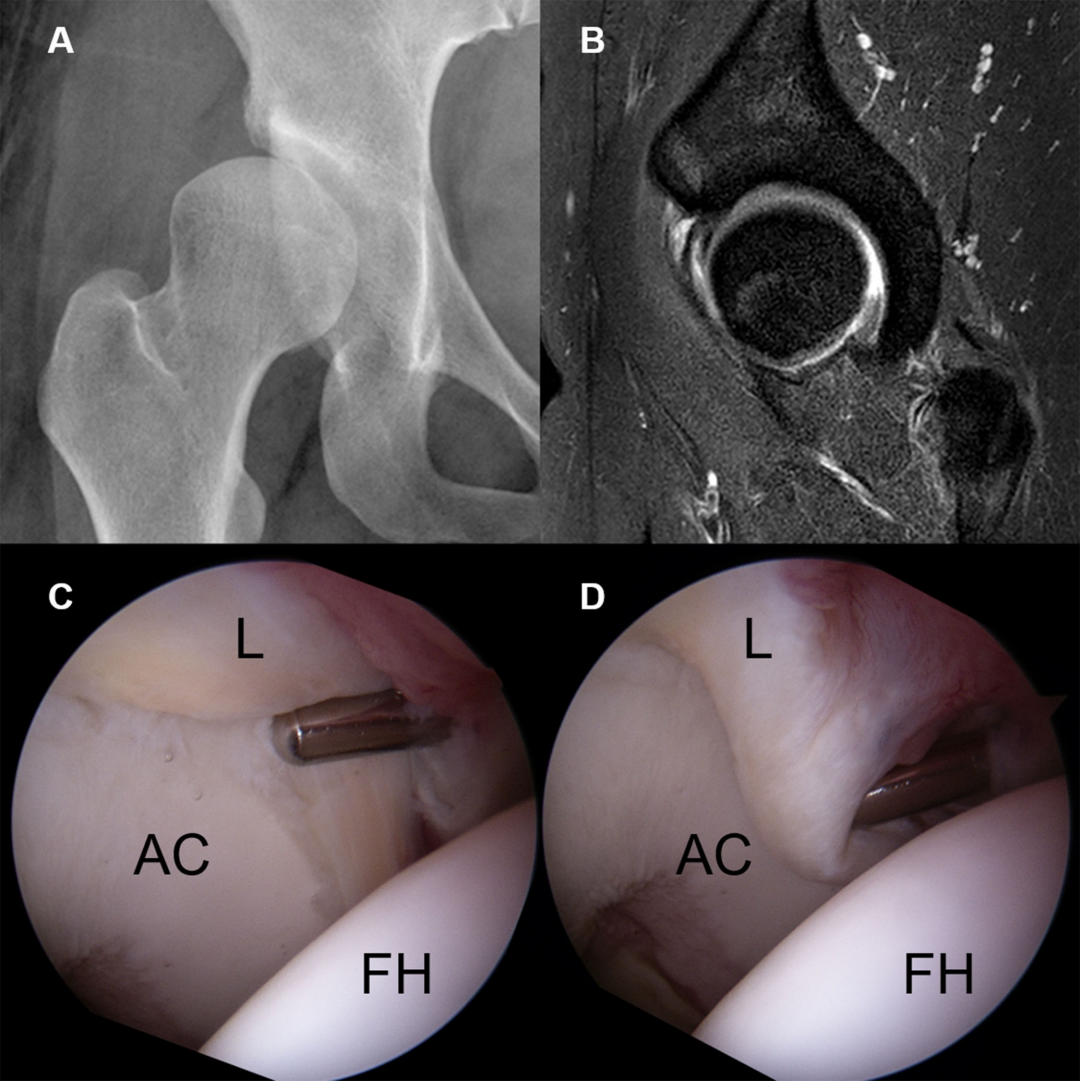
A grade 3 labral injury in a 26-year-old woman. (A) Simple X-ray showing a definite dysplastic acetabular structure. (B) Sagittal hip MRI showing increased intrasubstance signal intensity and a contrast material-filled paralabral cyst at the chondrolabral junction. (C, D) Arthroscopy showing an unstable labrum between the chondrolabral junction (C) and the capsulolabral recess (D), without labral displacement. (L, labrum; FH, femoral head; AC, acetabular cartilage).

**Fig 5:**
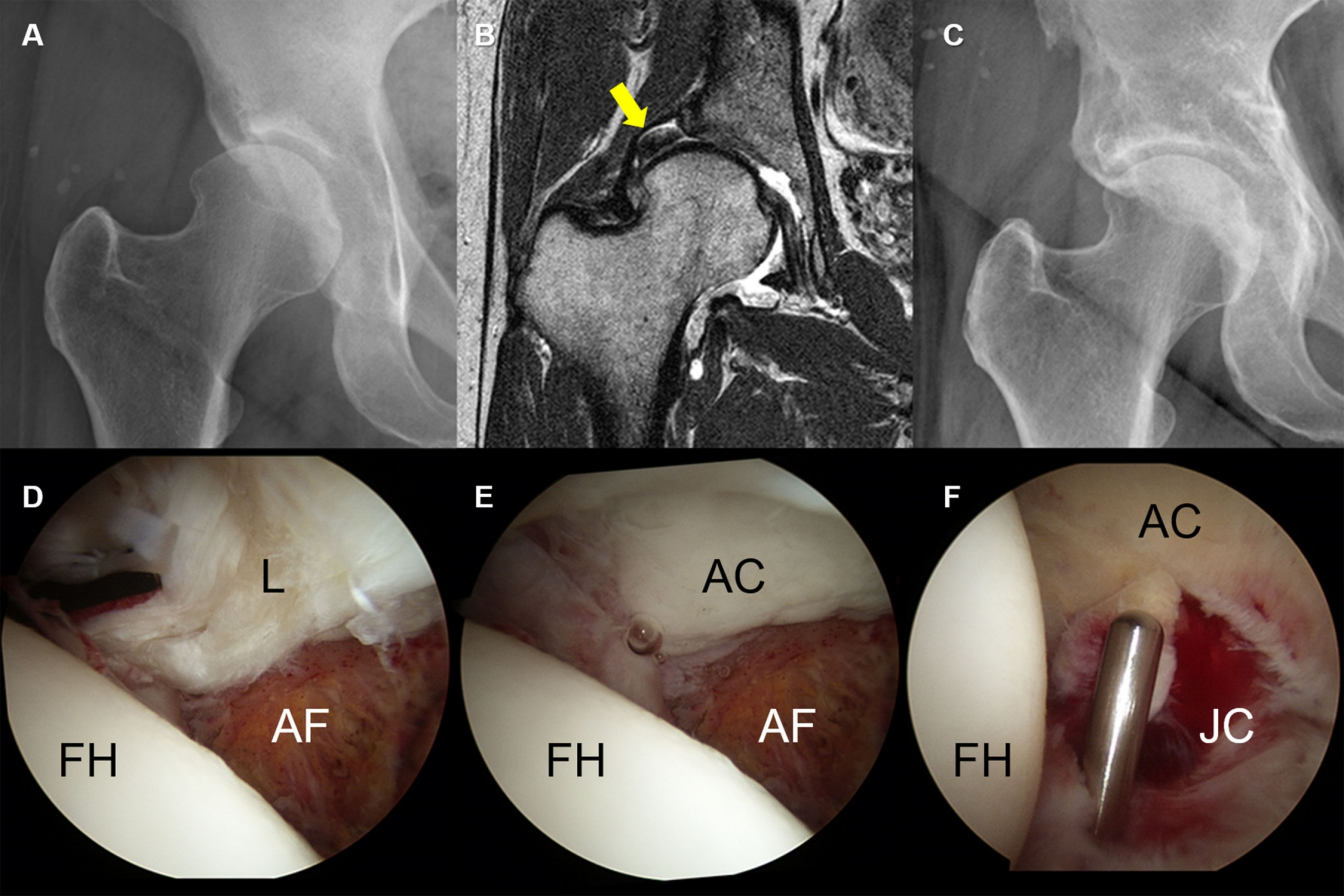
A grade 4 labral injury in a 45-year-old woman. (A) Simple X-ray showing joint space narrowing. (B). Coronal MRI image showing complete lateral displacement of the labrum (arrow). The patient underwent arthroscopy combined with PAO. (C) Follow-up X-ray at postoperative 3 years. (D, E) Arthroscopy showing labral displacement was shown as a bucket-handle tear. (F) Partial resection to remove an unstable degenerative labral tear. (L, labrum; FH, femoral head; AF, acetabular fossa; AC, acetabular cartilage; JC, joint capsule).

Seven (10.6%) labral tears were classified as Grade 1, characterized by partial delamination or blistering of the labrum with minimal fraying at the CLJ, with most having no definite labral pathology on MRI (Fig 2). Ten (15.2%) labral tears were classified Grade 2, involving only disruption of the CLJ. None showed CLR disruption, with the labrum being stable in all 10. MR arthrography showed contrast material-filled defects at the CLJ, indicating labral tears (Fig 3). Thirty (45.5%) labral tears were classified as Grade 3, as they showed the instability of the torn labrum with disruption of the CLJ and CLR. Because the torn labrum was not displaced, joint space width was maintained on AP radiographs. CLR disruption was observed on sagittal MRI as increased intrasubstance signal intensity or contrast material filling of the paralabral cyst at the CLR (Fig 4). Nineteen (28.8%) labral tears were classified as Grade 4, as they showed anterolateral displacement of the torn labrum with extensive disruption of the CLJ and CLR. Moreover, preoperative AP radiographs showed narrowing of the joint space (Fig 5). The details are shown in Table 1. The kappa coefficient was 0.72, indicating substantial interobserver agreement. Of the 66 tears, 45 (68.2%) were observed in zone 2 and 21 (31.8%) in zone 3.

**Table 1:** Arthroscopic classification of labral tears and numbers of patients with each type.

Type	Labrochondral junction	Capsulolabral recess	Labral stability	Patients n (%)	Combined PAO, n (%)
Total				66	30
1	Delamination	Intact	Stable	7 (10.6)	3/7 (42.9)
2	Disrupted	Intact	Stable	10 (15.2)	5/10 (50.0)
3	Disrupted	Disrupted	Unstable	30 (45.5)	7/30 (23.3)
4	Disrupted	Disrupted	Displaced	19 (28.8)	15/19 (78.9)

PAO, periacetabular osteotomy.

### Relationships between baseline characteristics with labral tear classification

Comparisons of baseline characteristics in the four tear grade groups showed that AD/WR was the only radiologic factor, and symptom duration was the only clinical factor, linearly related to tear grade (Table 2). Labral tear grade was negatively associated with AD/WR (*P* = 0.023) and positively associated with symptom duration (*P* = 0.017).

**Table 2:** Relationships between patient baseline characteristics and labral tear grade.

Variables	Mean ± SD (range)	P value
Mean LCEA (degree)		0.065
1	14.3 ± 5.7 (6 to 20)	
2	13.4 ± 4.5 (6 to 19)	
3	13.3 ± 5.8 (-3 to 24)	
4	10.9 ± 4.3 (6 to 20)	
Sharp angle (degree)		0.101
1	45.3 ± 2.6 (41 to 48)	
2	45.9 ± 3.5 (40 to 51)	
3	46.1 ± 4.6 (35 to 55)	
4	48.2 ± 4.7 (39 to 55)	
Tönnis angle (degree)		0.121
1	23.0 ± 5.7 (13 to 30)	
2	19.1 ± 5.6 (12 to 28)	
3	18.9 ± 6.4 (8 to 34)	
4	24.2 ± 5.9 (14 to 35)	
AD/WR		0.023
1	0.20 ± 0.03 (0.16 to 0.25)	
2	0.21 ± 0.39 (0.16 to 0.26)	
3	0.21 ± 0.03 (0.10 to 0.26)	
4	0.18 ± 0.03 (0.15 to 0.29)	
BMI (kg/m^2^)		0.078
1	23.3 ± 1.5 (20.7 to 24.9)	
2	24.4 ± 5.0 (19.1 to 36.0)	
3	23.5 ± 4.2 (18.1 to 34.6)	
4	25.9 ± 3.7 (21.6 to 35.3)	
Symptom duration (month)		0.017
1	16.3 ± 7.5 (7.1 to 29.3)	
2	25.0 ± 11.8 (11.2 to 43.2)	
3	31.7 ± 22.5 (7.9 to 85.2)	
4	40.4 ± 30.6 (18.0 to 108.2)	

Abbreviations: LCEA, lateral center edge angle; AD/WR, acetabular depth-to-width ratio; BMI, body mass index.

### Relationship between concomitant injuries and labral tear classification

The occurrence of concomitant injuries in hips classified by labral tear grade is shown in Table 3. Labral tear classification was found to correlate positively and linearly with FH damage (*P* < 0.001), acetabular cartilage damage (*P* < 0.001), and the incidence of paralabral cysts (*P* = 0.001). The incidence of complete LT tear did not correlate significantly with tear grade (*P* = 0.513).

**Table 3:** Relationships between concomitant injuries and arthroscopically determined labral tear grades.

Variables	n/N (%)	P value
Complete LT tear (21/66, 31.8%)		0.513
1	3/7 (42.9)	
2	1/10 (10.0)	
3	10/30 (33.3)	
4	7/19 (36.8)	
FH cartilage damage (23/66, 34.8%)		<0.001
1	2/7 (28.6)	
2	1/10 (10.0)	
3	5/30 (16.7)	
4	15/19 (78.9)	
Acet. cartilage damage (16/66, 24.2%)		<0.001
1	0/7 (0)	
2	0/10 (0)	
3	2/30 (6.7)	
4	14/19 (73.7)	
Paralabral cyst (37/66, 56.1%)		0.001
1	1/7 (14.3)	
2	2/10 (20.0)	
3	20/30 (66.7)	
4	14/19 (73.7)	

Abbreviations: LT, ligamentum teres; FH, femoral head; Acet., acetabulum.

### Treatment details following each labral tear classification

Of the seven hips classified as grade 1, three underwent simple labral debridement combined with PAO and four underwent all-arthroscopic labral base repair via mattress suture. Of the ten hips classified as grade 2, five underwent hip arthroscopy (simple labral debridement in two hips and labral repair in three) combined with PAO, and five underwent all-arthroscopic labral base repair. All grade 2 tears were repaired using the mattress suture technique, as were all 30 grade 3 labral tears. Of these 30 hips, seven also underwent PAO surgery, whereas nine of the 23 all-arthroscopically treated hips also underwent capsular plication. Of the 19 hips classified as grade 4, 15 underwent hip arthroscopy combined with PAO. Because labral repair could not be performed on eight of the 19 grade 4 hips, partial resection and marginal debridement were performed (Fig 5).

### Clinical outcomes

Mean mHHS scores were significantly higher (*P* < 0.001), whereas mean WOMAC scores were significantly lower (*P* < 0.001), after than before surgery. Although mean mHHS was higher and mean WOMAC score was lower postoperatively than preoperatively in patients with all four labral tear grades, none of these differences was statistically significant (Tables 4 and 5).

**Table 4:** Relationships of preoperative and final postoperative modified Harris hip scores with labral tear grades.

Gr	Preoperative mHHS			Final mHHS		
	A/S only	w/ PAO	Total	A/S only	w/ PAO	Total
1	65.7 (SD 12.9)	82.0 (SD 7.1)	71.5 (SD 15.2)	78.0 (SD 12.7)	89.7 (SD 2.3)	83.5 (SD 9.8)
2	55.5 (SD 4.4)	86.0 (SD 7.1)	62.6 (SD 16.3)	67.8 (SD 13.5)	87.2 (SD 3.8)	71.8 (SD 14.8)
3	59.1 (SD 13.2)	68.3 (SD 20.1)	58.6 (SD 15.9)	72.8 (SD 13.8)	83.9 (SD 9.0)	73.8 (SD 14.5)
4	59.0 (SD 19.2)	61.0 (SD 14.9)	57.9 (SD 16.5)	64.8 (SD 17.4)	77.6 (SD 13.0)	59.4 (SD 17.5)

Abbreviations: Gr, grade; mHHS, modified Harris hip score; A/S, arthroscopy; PAO, periacetabular osteotomy.

**Table 5:** Relationships of preoperative and final postoperative WOMAC scores with labral tear grades.

Gr	Preoperative WOMAC			Final WOMAC		
	A/S only	w/ PAO	Total	A/S only	w/ PAO	Total
1	24.3 (SD 12.5)	10.5 (SD 7.8)	20.5 (SD 13.5)	22.5 (SD 24.7)	2.0 (SD 3.5)	12.8 (SD 18.4)
2	51.0 (SD 10.9)	19.7 (SD 16.9)	41.0 (SD 24.3)	19.2 (SD 16.0)	5.4 (SD 6.6)	19.2 (SD 16.0)
3	23.4 (SD 14.7)	31.3 (SD 23.9)	26.9 (SD 17.9)	20.9 (SD 16.0)	18.4 (SD 20.8)	18.2 (SD 20.2)
4	27.8 (SD 23.1)	30.7 (SD 5.8)	29.0 (SD 16.8)	14.8 (SD 11.3)	13.6 (SD 12.0)	19.0 (SD 13.9)

Abbreviations: Gr, grade; A/S, arthroscopy; PAO, periacetabular osteotomy; WOMAC, Western Ontario and McMaster Universities Osteoarthritis index.

Three hips, all with grade 4 labral tears, required conversion to total hip arthroplasty (THA). Of these, two underwent hip arthroscopy alone, and one underwent arthroscopy plus PAO at index surgeries. These hips had a mean LCEA of 11.3° ± 3.5° (range 8° to 15°), a mean Sharp angle of 43.7° ± 4.2° (range 39° to 47°), a mean Tönnis angle of 24.3° ± 1.5° (range 23° to 26°), and a mean AD/WR of 0.18 ± 0.02 (range 0.16 to 0.20). No patient required revision arthroscopy.

### Preoperative & final Tönnis OA grades

Table 6 shows Tönnis OA grades preoperatively and at final follow-up. Preoperatively, 63 of the 66 hips were classified as having Tönnis grade 0 or 1; at final follow-up, however, 13 hips were classified as having Tönnis grade 2 or 3. The OA progression to Tönnis grade 2 or 3 was most common in hips with grade 4 labral tears. Moreover, Spearman correlation analysis showed a positive linear relationship between preoperative OA grade and labral tear grade (*P* < 0.001, rho = 0.560).

**Table 6:** Relationships between preoperative and final postoperative Tönnis OA grades and labral tear grades.

Tear grade	Preoperative Tönnis OA grade				Final postoperative Tönnis OA grade			
	Gr 0 (n, %)	Gr 1 (n, %)	Gr 2 (n, %)	Gr 3 (n, %)	Gr 0 (n, %)	Gr 1 (n, %)	Gr 2 (n, %)	Gr 3 (n, %)
1	6 (85.7)	1 (14.3)	0	0	5 (71.4)	2 (28.6)	0	0
2	9 (90.0)	1 (10.0)	0	0	4 (40.0)	6 (60.0)	0	0
3	20 (66.6)	10 (33.3)	0	0	8 (26.7)	16 (53.3)	6 (20.0)	0
4	3 (15.8)	13 (68.4)	3 (15.8)	0	0	6 (31.6)	7 (36.8)	6 (31.6)

Abbreviations: OA, osteoarthritis; Gr, grade.

## Discussion

The current study proposes a new grading system for labral tears in patients with hip dysplasia. This new grading system showed a positive linear relationship with symptom duration and a negative relationship with AD/WR. Tear severity correlated positively and linearly with FH damage, acetabular cartilage damage, and the incidence of paralabral cysts.

Acetabular labral tears have been classified as radial flap, radial fibrillated, longitudinal peripheral, and unstable types, based on arthroscopic findings [5], similar to the classification of meniscal injuries of the knee. Although this classification system is used most frequently for the categorization of hip labral tears [12], it is not sufficient for describing labral tears in patients with acetabular dysplasia and it shows poor correlation with the results of MR arthrography. Labral tears have also been classified based on MR arthrography [4], although this classification is inadequate for treatment planning, as it only highlights the visualization of the labrum itself, without addressing the recess located between the joint capsule and the labrum. Acetabular labral tears have also been histologically classified into two types based on a study in cadavers, with one type consisting of a detachment of the labrum from the articular cartilage surface, and the other consisting of one or more cleavages within the substance of the labrum [7]. That study was the first to investigate the histological features and microvasculature structure associated with labral tears; however, it did not address the clinical findings of each type and did not fit labral tears in acetabular dysplasia. A scoring system for hip labral tears not specific to dysplastic hips and based on MR arthrography was introduced in 2007 [13]. That study reported an interrelationship between labral tears and cartilage damage, comparable with the results of the present study, which showed a positive linear relationship between the severity of tear grading and FH/acetabular cartilage damage. The recently introduced Mahorn classification system includes evaluations of the sizes of labral tears, tear patterns, and intrasubstance changes [14]. All of these classification systems, however, have limitations, including the lack of consideration of injury mechanism and of a systematic staged approach. In particular, labral tears in some patients with acetabular dysplasia cannot be classified using previous criteria. An MRA-based scoring system of labral tears in patients with FAI showed that higher scores for tear extension and thickness were more frequently associated with debridement or suture of the labrum [15]. Indeed, the labrum of patients with hip dysplasia is usually hypertrophied, making more tissue amenable to repair and increasing the predisposition to degenerative changes. Moreover, in the current study, all patients with grade 4 tears underwent partial resection and marginal debridement procedures.

**Fig 6:**
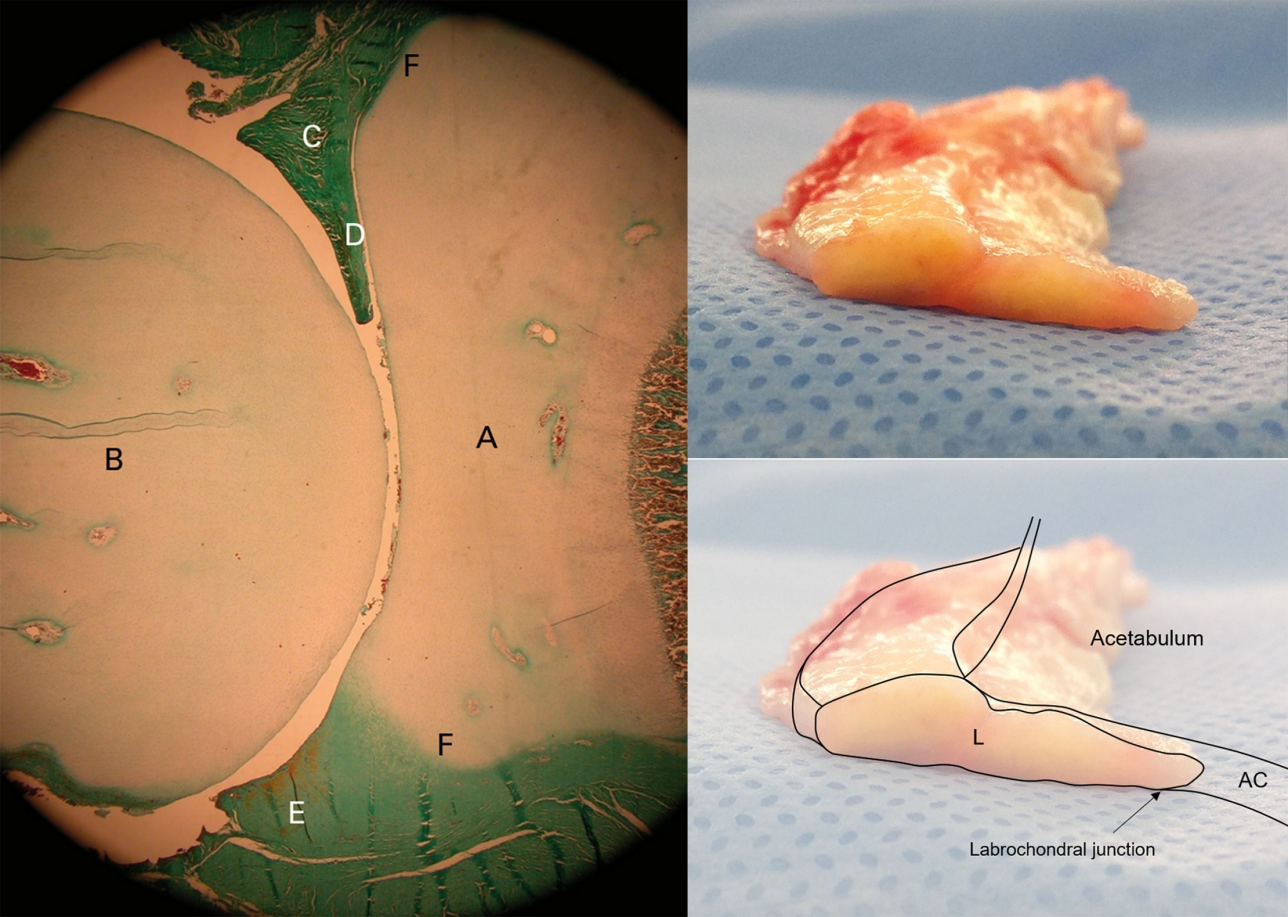
Photomicrograph of a fetal hip at term (left, reprinted with permission from M. Cashin et al.: Embryology of the acetabular labral-chondral complex. J Bone Joint Surg [Br] 2008;90-B:1019–24. Copyright 2008 Springer.) (A, acetabulum; B, femoral head; C, anterior labrum; D, intra-articular projection of the anterior labrum; E, posterior labrum; F, acetabular labral transition zone). Gross specimen of a labrum (right top), resected during total hip arthroplasty from a patient with hip dysplasia, and a schematic drawing of the acetabulum including it (L, labrum; AC, acetabular cartilage).

In normal hip joints, the acetabular labrum merges with the articular cartilage through a 1–2 mm transition zone and attaches firmly to the articular side of the consistent thin tongue of the bone that extends from the edge of the bony acetabulum via a zone of calcified cartilage [7]. Although labral enlargement may compensate for the relative lack of acetabular bony coverage to maintain the FH within the joint in dysplastic hips, the transition zone is also a weak point and is vulnerable to injury, especially in the anterior segment [16]. An embryological study of the acetabular labral-chondral complex reported a marginal attachment of the anterior labrum to the acetabular cartilage with an intra-articular projection [17]. Inspection of a gross specimen of the labrum, which was resected during the THA due to the progression of osteoarthritis caused by hip dysplasia, showed an intra-articular portion similar to the embryological intra-articular projection (Fig 6). An intra-articular projection with a sharper and more abrupt transition zone of the anterior labral-chondral complex, compared with the posterior, may make the anterior labrum more prone to tearing. In dysplastic hips lacking bony coverage, this intra-articular portion of the labrum is essential to maintain the femoral head within the joint. In the current study, joint space narrowing on preoperative AP radiographs was observed in all hips with grade 4 labral tears, which showed anterolateral displacement of the torn labrum with extensive disruption of the CLJ and CLR. Therefore, if possible, the intra-articular portion of the torn labrum in dysplastic hips should be preserved with limited debridement during labral repair.

In the current study, AD/WR showed a significant negative relationship with tear grade, whereas most patient demographic characteristics, such as mean LCEA, Sharp angle, Tönnis angle, and BMI, were unrelated to tear grade. However, symptom duration and Tönnis OA grade showed significant positive linear relationships with tear grade. These findings suggest that the mechanisms of injury associated with the various tear grades did not differ, as they may follow the natural course of labral tear and severity. This hypothesis is supported by our classification system, which matches the progression of labral tears in dysplastic hips.

The incidence of concomitant injuries of labral tears in acetabular dysplasia was assessed by investigating the occurrence of LT tears, FH and acetabular cartilage damage, and paralabral cysts. These concomitant injuries frequently accompany labral tears in patients with hip dysplasia [1, 18–20], but none of the previous classification systems considered them factors related to labral tears. Except for LT tears, all of these injuries showed a positive linear relationship with labral tear grade. Although an extended study with a larger sample size is necessary to define this relationship, the results obtained here suggest that LT tears occur earlier than the other injuries investigated in patients with hip dysplasia and labral tears.

Our grading system also suggests a treatment plan. Patients classified as having grade 1 and 2 injuries were treated by simple debridement, with or without labral base repair. All hips classified as grade 3 underwent labral repair, whereas grade 4 hips were treated with labral repair or partial labral resection. Labral base repair was attempted, when possible. In all groups, mean mHHS was higher and mean WOMAC score lower at final follow-up than preoperatively, indicating that treatment was successful, although the differences were not statistically significant.

Interestingly, patients with grade 4 tears showed an improvement in mHHS scores of only 1.5 points. Moreover, three patients initially classified with grade 4 labral tears required THA conversion. Also, a comparison of preoperative and final follow-up OA grades showed no progression to Tönnis grade 2 or 3 in patients with grade 1 / 2 labral tears. However, 20% of patients with grade 3 tears progressed to Tönnis grade 2 OA and almost 70% of those with grade 4 labral tears progressed to Tönnis grade 2 or 3 at final follow-up period. Thus, arthroscopic treatment should be carefully considered for grade 4 labral tears in dysplastic hips.

This study had several limitations. First, the sample size was small, which may have affected the results. Second, we could not provide imaging correlations for all patients, due to the retrospective nature of this study and the inability to standardize the imaging modality. Although all patients underwent MRI or MR arthrography, the series could not be standardized, and we could not confirm the radiological reports, especially for images obtained from another hospital. Third, the performance of combined PAO surgery was not randomized because hip arthroscopy was the assisting procedure in patients with initially planned PAO. Additional studies using a larger number of patients are necessary to confirm the results reported here and to correlate them with imaging data.

In conclusion, arthroscopic findings of labral tears in patients with hip dysplasia differed from the conventional classification. The classification system proposed in this study will likely be useful for determining the degree of labral tear in patients with hip dysplasia and for predicting treatment outcomes.
